# Analysis of the Microbial Community in an Acidic Hollow-Fiber Membrane Biofilm Reactor (Hf-MBfR) Used for the Biological Conversion of Carbon Dioxide to Methane

**DOI:** 10.1371/journal.pone.0144999

**Published:** 2015-12-22

**Authors:** Hyun Chul Shin, Dong-Hun Ju, Byoung Seung Jeon, Okkyoung Choi, Hyun Wook Kim, Youngsoon Um, Dong-Hoon Lee, Byoung-In Sang

**Affiliations:** 1 Clean Energy Research Center, National Agenda Research Division, Korea Institute of Science and Technology (KIST), Seongbuk-gu, Seoul, South Korea; 2 Department of Environmental Engineering, University of Seoul, Dongdaemun-Ku, Seoul, South Korea; 3 Department of Chemical Engineering, Hanyang University, Seongdong-Ku, Seoul, South Korea; 4 The Research Institute of Industrial Science, Hanyang University, Seongdong-Ku, Seoul, South Korea; Medical University Graz, AUSTRIA

## Abstract

Hydrogenotrophic methanogens can use gaseous substrates, such as H_2_ and CO_2_, in CH_4_ production. H_2_ gas is used to reduce CO_2_. We have successfully operated a hollow-fiber membrane biofilm reactor (Hf-MBfR) for stable and continuous CH_4_ production from CO_2_ and H_2_. CO_2_ and H_2_ were diffused into the culture medium through the membrane without bubble formation in the Hf-MBfR, which was operated at pH 4.5–5.5 over 70 days. Focusing on the presence of hydrogenotrophic methanogens, we analyzed the structure of the microbial community in the reactor. Denaturing gradient gel electrophoresis (DGGE) was conducted with bacterial and archaeal 16S rDNA primers. Real-time qPCR was used to track changes in the community composition of methanogens over the course of operation. Finally, the microbial community and its diversity at the time of maximum CH_4_ production were analyzed by pyrosequencing methods. Genus *Methanobacterium*, related to hydrogenotrophic methanogens, dominated the microbial community, but acetate consumption by bacteria, such as unclassified *Clostridium* sp., restricted the development of acetoclastic methanogens in the acidic CH_4_ production process. The results show that acidic operation of a CH_4_ production reactor without any pH adjustment inhibited acetogenic growth and enriched the hydrogenotrophic methanogens, decreasing the growth of acetoclastic methanogens.

## Introduction

Most methanogens convert carbon dioxide (CO_2_) to methane (CH_4_), the major flammable component of natural gas. CH_4_ can be used to make a renewable, carbon-neutral gas substitute [1–3]. Hydrogenotrophic methanogens can upgrade CO_2_ to CH_4_ using molecular hydrogen (H_2_) via a process referred to as biomethanation [4]. Previous studies have shown that hydrogenotrophic methanogens were enriched at a relatively short retention time (1.25 days) [5]. High temperature supported the growth of hydrogenotrophic methanogens due to the presence of active thermophilic methanogens [6, 7]. Another study showed that hydrogenotrophic methanogens were dominant after the long-term cultivation of a psychroactive methanogenic community at 4–10°C [8]. Hydrogenotrophic methanogens were also found in extreme conditions, such as acidic peat [9]. Therefore, the advantages of hydrogenotrophic biomethanation, including biogas upgrading [10], its high CO_2_ → CH_4_ conversion ratio [11], and its tolerance to environmental perturbation in the field [12], can be used in anaerobic digestion under various conditions, such as in acidogenic reactors [9, 13].

The optimization of CH_4_ production by hydrogenotrophic methanogens has been studied by controlling the gassing rate [14, 15], the reactor pressure [14, 16], and reactor design [17] with hydrogenotrophic methanogens in pure culture, such as *Methanothermobacter marburgensis* [14]. In this study, we studied a microbial community from wastewater treatment sludge that was capable of converting CO_2_ to CH_4_ (conversion ratio, 90%) by biomethanation using CO_2_ and H_2_ with a hollow-fiber membrane biofilm reactor Hf-MBfR. Our hypothesis was that the Hf-MBfR could properly supply H_2_ and the hydrogenotrophic methanogen could use H_2_, preventing its release to air. To study the changes of hydrogenotrophic methanogen community structure, a time series of collected biomass samples was analyzed using Denaturing Gradient Gel Electrophoresis (DGGE), and the enriched microbial community was investigated using pyrosequencing using primers targeting the V1 to V3 regions of the 16S rRNA gene. Taxonomic quantification was performed using quantitative PCR (qPCR) targeting generic bacterial and archaeal sequences, as well as *Methanobacteriales* (hydrogenotrophic methanogens) and *M*ethanomicrobiales (acetoclastic methanogens).

## Materials and Methods

### Hollow-fiber membrane biofilm reactor (Hf-MBfR)

**Fig 1:**
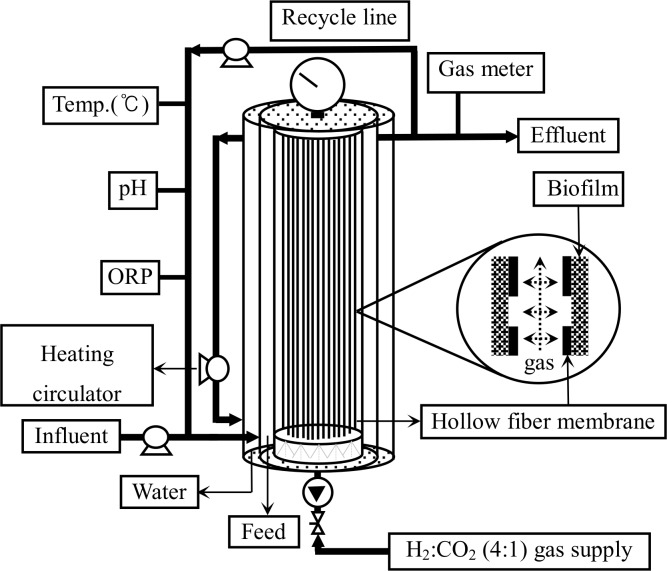
Schematic diagram of Hollow Fiber Membrane Biofilm Reactor (Hf-MBfR) system [11].

Fig 1 shows the schematic diagram of the Hf-MBfR (Chemicore Co., Ltd.) used in this study. The reactor was operated at acidic conditions (pH 4.5–5.5) without any pH control; the fiber inner and outer diameters of 1.4 mm, 0.8 mm, respectively; the total volume was 330 mL; working volume was 195 mL; the recirculation rate was and 10 mL/min [11]. The reactor was maintained in anaerobic condition by purging mixed gases (H_2_:CO_2_ = 4:1) through the hollow-fiber membrane and by keeping the inner reactor temperature at 35–38°C with a heating circulator. The supplied mixed gases were used as substrates for the biological conversion of CO_2_ to CH_4_. The pressure of the mixed gases was 1.4–2.1 kPa. Sieved anaerobic digested sludge (initial inoculum, **S1 Fig**) collected from a wastewater treatment plant (Jungrang wastewater treatment plant, Seoul) was inoculated into each reactor at a level of 20% of the working volume. The mineral medium composition of the reactor is shown in Table 1. The inner reactor was mixed with the up-flow using a recycling pump. ORP (oxidation-reduction potential), temperature, and pH were continuously observed with an ORP probe, a thermometer, and a pH meter, respectively. A wet gas-meter (Model W-NK-0.5, Shinagawa, Japan) was used to measure the volume of gases produced. Gases taken from the sampling port installed at the gas effluent line were analyzed by GC-TCD, and volatile organic acid analysis was performed by GC-FID.

**Table 1:** Mineral medium composition of the reactor for microbial CO_2_ reduction by methanogens.

Compounds	Concentration(mg/L)
MgCl_2_6H_2_O	16.05
CaCl_2_2H_2_O	1.20
ZnCl_2_	5.91
Na_2_Mo2H_2_O	1.29
MnCl_2_4H_2_O	13.19
CuCl_2_2H_2_O	2.61
CoCl_2_6H_2_O	0.3
KCl	1.00
FeCl_2_2H_2_O	5.23
EDTA	9.75
NaCl	200
(NH_4_)_2_PO_4_	200

### Total genomic DNA extraction

Samples (3–5 mL) were taken at the recycling line for microbial community analysis. After centrifugation, the supernatant was collected for volatile organic acids analysis, and the pellet was used for the extraction of total genomic DNA with a Power Soil DNA Kit (MO BIO, Carlsbad, USA). The quality of the extracted DNA was examined with standard agarose gel electrophoresis and stored at -20°C. The extracted DNA was used as the template for the 16S rDNA PCR.

### Bacterial and archaeal 16S rDNA PCR-DGGE

PCR for microbial community analysis was conducted with nested PCR. The 1^st^ PCR amplification of the bacterial 16S rRNA genes was performed with primers 27f and 1492r, and with primers 46f and 1100r for archaeal 16S rRNA genes. The PCR amplification was performed in 50 μl reaction mixtures containing 5μl Ex 10× PCR buffer (Takara, Japan), 8 μl 2.5 mM dNTPs, 2 μl each primer (20 pmol/μl), 2.5U of Ex Taq polymerase (Takara, Japan), 2 μl template DNA and 30.5 μl distilled water. The thermo cycling program was: 5 min initial denaturation at 95°C (94°C for 2 min for archaeal DNA), followed by 30 cycles of 1 min at 94°C, 1 min at 58°C (57°C for 1 min), and 1 min at 72°C, followed by 10 min of final extension at 72°C. The 2^nd^ PCR amplification of the 16S rRNA genes was performed with primers with the primer pairs 341f-GC/534r and 340f-GC/519r for bacterial and archaeal DNA, respectively. The PCR product contained a GC clamp of 40 bases, added to the forward primer and had a total length of 233 bp, including the highly variable V9 region. PCR reactions were prepared in 50 μl reaction mixtures containing 2x GCⅡ buffer (Takara, Japan). For bacterial DNA, the PCR cycles consisted of an initial denaturation at 94°C for 5 min, followed by 10 cycles of 94°C for 30 sec, 63°C for 30 sec, and 72°C for 30 sec, followed by 20 cycles of 94°C for 30 sec, 58°C for 30 sec, and 72°C for 30 sec, followed by a final extension at 72°C for 10 min. For archaeal DNA, the PCR cycles consisted of an initial denaturation at 95°C for 10 min, followed by 20 cycles of 94°C for 30 sec, 72°C (-0.5°C/cycle) for 30 sec, and 72°C for 1 min, followed by 20 cycles of 94°C for 30 sec, 62°C for 30 sec, and 72°C for 1 min, followed by a final extension at 72°C for 10 min. Each PCR sample was checked by electrophoresis on horizontal 1.2% agarose gels and purified from agarose gel slices with a QIAquick Gel Extraction Kit (QIAGEN, Valencia, USA). The PCR products were subjected to DGGE with the Dcode^TM^ Universal Mutation Detection System (BIO-RAD, Hercules, USA) and run on 10% (wt/vol) polyacrylamide gels with a denaturing gradient, ranging from 25% to 55% for bacterial DNA, and from 40% to 55% for archaeal DNA. The gels were electrophoresed for 14 hr at 60°C at a constant voltage of 60 V after electrophoresis at 20 V for 20 min. After electrophoresis, the gels were stained with ethidium bromide (EtBr) for 15 min, rinsed for 10 min, and photographed with UV transillumination (302 nm).

For sequencing, the selected bands were excised from the DGGE gels using a sterile scalpel and placed in a sterile Eppendorf tube containing 40 μl of sterile water, and the DNA was eluted using five cycles of freeze-thawing (-70°C/37°C). Two microliters of the solution were used as template DNA in the PCR using the 2^nd^ bacterial and archaeal PCR protocol with non-GC clamp primers. The amplified products were purified from agarose gel slices with a QIAquick Gel Extraction Kit (QIAGEN, Valencia, CA). The purified PCR products were cloned with the yT&A vector cloning kit (Yeastern Biotech, Taipei, Taiwan) following the manufacturer’s instructions. Two or three white colonies were randomly picked from each cloned sample for plasmid isolation (Nucleogen, Daejeon, South Korea). Sequencing was performed with an ABI Prism BigDye Terminator Cycle Sequencing Ready Kit (Applied Biosystems, Foster City, USA) according to the manufacturer's instructions with the sequencing primer M13f for the vector. Approximately 200 unambiguous nucleotide positions were used for comparison with the data in GenBank using the Basic Local Alignment Search Tool (BLAST). Sequences from the nearest relatives were identified from BLAST.

### Real-time qPCR for bacteria and archaea

The DNAs extracted from the samples of the reactors were used to construct standard curves for bacteria, archaea, *Methanobacteriales* and *Methanomicrobiales*. First, the PCR results using each 16S rRNA primer (Table 2) were used to determine the copy number after transformation for each taxonomic group. The PCR amplification was performed in 25 μl reaction mixtures containing 2.5μl Ex 10× PCR buffer (Takara, Japan), 4 μl 2.5 mM dNTPs, 2.5 μl of each primer (9 pmol/μl), 1.25 U Ex Taq polymerase (Takara, Japan), 1 μl of template DNA and 12.25 μl of distilled water. The PCR cycles consisted of an initial denaturation at 95°C for 10 min, followed by 30 cycles of 95°C for 30 sec, with annealing temperatures of 58°C, 60°C, 60°C, and 63°C for bacteria, archaea, *Methanobacterialses*, and *Methanomicrobiales*, respectively, for 1 min, and 72°C for 30 sec and a final extension at 72°C for 10 min. The 16S rRNA genes with proper sizes were extracted and purified with a QIAquick Gel Extraction Kit (QIAGEN, Valencia, USA). Each purified DNA was cloned with the yT&A vector cloning kit (Yeastern Biotech. Taipei, Taiwan) following the manufacturer’s instructions. Transformed colonies were incubated in LB broth (1% tryptone, 0.5% NaCl, and 0.5% yeast extract) at 37°C for 16 h. The culture solution was centrifuged, and plasmids were obtained from the pellet. PCR was performed to obtain a high concentration of DNA as described above, using these plasmids as a template. The concentrations of the PCR products were measured with a BIORAD VersaFluor™ fluorometer. By assuming an average molecular weight of 660 Da for a base pair in double-stranded DNA [18], the following equation (Eq 1) was used to calculate the number of 16S rRNA gene copies that were present in the obtained DNA [19].

**Table 2:** Primer sets used in this study for PCR-DGGE.

Primers^a^	Primer sequence (5’ to 3’)	Specificity
27f	GAG TTT GAT CMT GGC TCA G	Bacteria
1492r	GGY TAC CTT GTT ACG ACT T	
341f^b^	CCT ACG GGA GGC AGC AG	
534r	ATT ACC GCG GCT GCT GG	
PRA46f	YTA AGC CAT GCR AGT	Archaea
PREA1100r	YGG GTC TCG CTC GTT RCC	
PARCH340f^c^	CCC TAC GGG GYG CAS CAG	
PARCH519r	TTA CCG CGG CKG CTG	

^a^ f, forward primer; r, reverse primer.

^b^ GC clamp sequence for 341f, 5’-CGC CCG CCG CGC CCC GCG CCC GTC CCG CCG CCC CCG CCC G-3’attached to 5’ end of the primer

^c^ GC clamp sequence for PARCH340f, 5’-CGC CCG CCG CGC GCG GCG GGC GGG GCG GGG GCA CGG GGG G-3’ attached to 5’ end of the primer

$$16\text{S rDNA}\;(\text{copy}/\text{mL})=\frac{16S\;rDNA\;concentration\;(g/ml)\times 6\times 10^{23}}{16S\;rDNA\;amplicon\;size\;(bp)\times 660(g\;16S\;rDNA/mol/bp)}$$ (1)

Initial 16S rRNA gene copy numbers ranged on the order of 10^11^ for each target, and they were serially diluted. The diluted samples and unknown samples were amplified with a real-time qPCR system (Applied Biosystems 7300 Real Time PCR System) with each primer and probe set (Table 3). Real-time qPCR was performed in 25 μl reaction mixtures containing 12.5μl 2×PCR master mix (Promega, Fitchburg, USA), 2.5 μl of each primer (9 pmol/μl) and probe (2.5 pmol/μl), 1 μl template DNA and 4 μl distilled water. PCR cycles consisted of 50°C for 2 min and an initial denaturation at 95°C for 10 min, followed by 40 cycles of 95°C for 15 sec, and then a combined annealing/extension step at 60°C for 1 min (58°C for bacteria). Logarithmic values of the different 16S rRNA gene amounts were plotted against the threshold cycle (*C*_T_) numbers from each qPCR assay. The linear ranges of the standard curves were selected based on the *r* ^2^ of the slope being greater than 0.995 except for *Methanomicrobiales* (0.930).

**Table 3:** Primer and probe sets used in this study for qPCR assay.

Primers^a^	Primer sequence (5’ to 3’)	Specificity
1055F	ATG GCT GTC GTC AGC T	Bacteria
1392R	ACG GGC GGT GTG TAC	
16STaz1115	6FAM-CAA CGA GCG CAA CCC -TAMRA	
787F	ATT AGA TAC CCS BGT AGT CC	Archaea
1059R	GCC ATG CAC CWC CTC T	
915F	6FAM-AGG AAT TGG CGG GGG AGC AC-TAMRA	
857F	CGW AGG GAA GCT GTT AAG T	*Methanobacteriales*
1196R	TAC CGT CGT CCA CTC CTT	
929F	6FAM-AGC ACC ACA ACG CGT GGA-TAMRA	
282F	ATC GRT ACG GGT TGT GGG	*Methanomicrobiales*
832R	CAC CTA ACG CRC ATH GTT TAC	
749F	6FAM-TYC GAC AGT GAG GRA CGA AAG CTG-TAMRA	

### Pyrosequencing

Upon attainment of maximum CH_4_ production, total DNA was extracted with the Power Soil^TM^ DNA isolation kit (MO BIO, Carlsbad, USA), according to the manufacturer’s instructions. The 16S rRNA genes were amplified (Roche 454 GS FLX Titanium) using bar-coded universal primers for each sample. The primer sequences were as follows: bacterial universal (27F: AGA GTT TGA TCM TGG CTC AG, 518r: WTT ACC GCG GCT GCT GG) and archaeal universal (arc112F: GCT CAG TAA CAC GTG G, arc516r: GGT DTT ACC GCG GCK GCT G) for bacterial and archaeal 16S rRNA gene amplification, respectively.

The amplifications was carried out under the following conditions: initial denaturation at 95°C for 5 min, followed by 30 cycles of denaturation at 95°C for 30 sec, primer annealing at 55°C for 30 sec, and extension at 72°C for 30 sec, followed by a final elongation at 72°C for 5 min. The amplified products were purified with the QIAquick PCR purification kit (Qiagen, Valencia, USA). Obtained reads from the different samples were sorted by the unique barcodes of each PCR product. The sequences of the barcode, linker, and primers were removed from the original sequencing reads. Potential chimera sequences were detected with Bellerophon, which involves comparing the BLASTN search results between the forward and reverse half-sequences [20]. Reads were assigned against the EzTaxon-e database (http://eztaxon-e.ezbiocloud.net) [21], which contains 16S rRNA gene sequences from type strains that have valid published names and representative species level phylotypes of either cultured or uncultured entries in the GenBank database with complete hierarchical taxonomic classification from the phylum to the species. The term uc means “unclassified taxon,” and typical suffixes are _s (for species), _g (genus), _f (family), _o (order), _c (class) and _p (phylum) [22]. The results obtained from archaeal and bacterial communities, as well as from the different analysis methods, were compared with each other. From the pyrosequencing analysis, 15,043 bacterial sequence reads and 40,766 archaeal sequence reads were acquired. Low-quality and chimeric sequences were removed. The average read lengths for archaea and bacteria were 399 bp and 453 bp, respectively. To compare OTUs between samples, shared OTUs were obtained with the XOR analysis of the CLcommunity program (Chunlab Inc., Seoul, South Korea). Interactive Krona HTML5 [23] hierarchical and double pie chart community profiles have been included in the supplemental information online as charts_supplemental.zip (**S1 File**). Sequences from this study were deposited in the NCBI short-read archive under the accession number SRA051716.

## Results and Discussion

### Bacterial 16S rDNA PCR-DGGE

**Fig 2:**
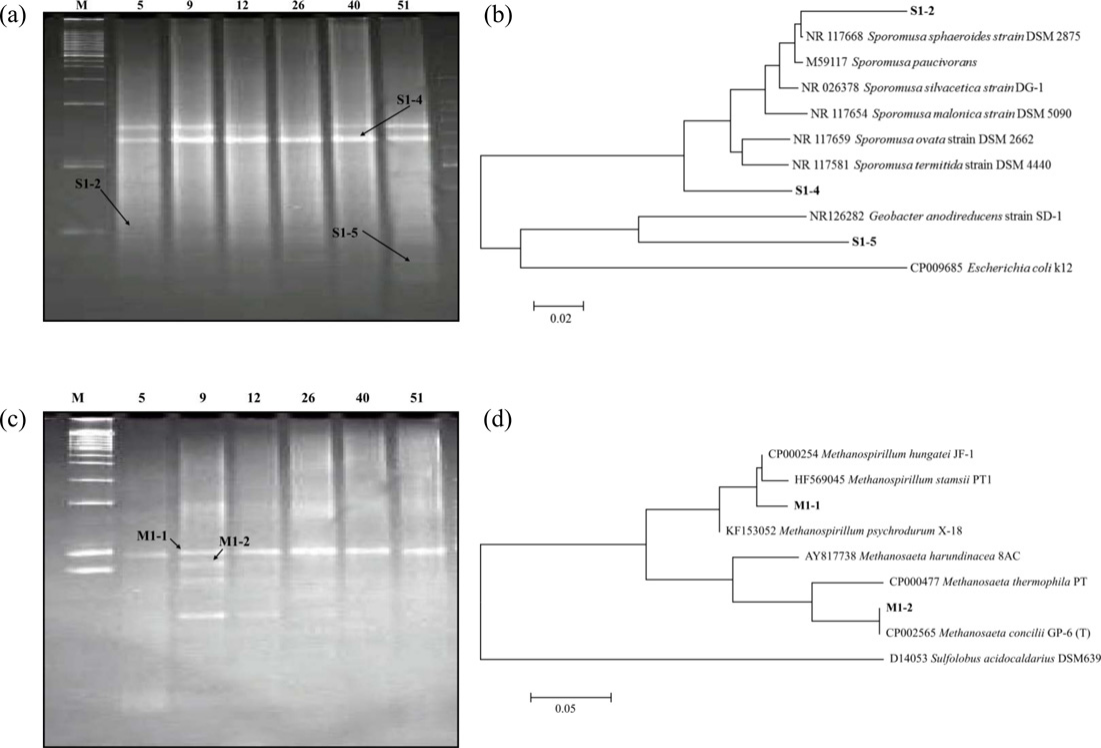
The profile of 16S rDNA PCR-DGGE for (a) bacteria and (c) archaea in Hf-MBfR. The line number indicates the sampling hour and ‘M’ represents a marker. This neighbor-joining tree shows the relationships between representative sequences and their related strains (b, bacteria; d, archaea). *Escherichia coli* K12 (CP009685) and *Sulfolobus acidocaldarius* DSM 639T (CP000077) were used as the outgroup for bacteria and archaea, respectively. Scale bar: 0.02 nucleotide substitutions per position for bacteria and 0.05 for archaea.

Fig 2 shows the bands of the bacterial 16S rDNA PCR-DGGE (a) and neighbor-joining tree of representative bacterial sequences showing the relationships between representative sequences and their related strains (b). The band intensity of the major *Sporomusa* strain (S1-4) increased with the length of the fermentation period (Fig 2A). *Sporomusa malonica* is a Gram-negative spore-forming homoacetogen [24], and *Sporomusa sphaeroides* is a Gram-negative, spore-forming, banana-shaped bacteria with a described pH-range between 5.7 and 8.7 [25]. *Sporomusa termida* sp. nov., is an H_2_/CO_2_-utilizing acetogen isolated from termites [26], and *Sporomusa paucivorans* sp. nov., a methylotrophic bacterium that forms acetic acid from H_2_ and CO_2_ [27]. The *Sporomusa* sp. identified as a result of the DGGE band sequencing was found to be an acetogen. It was suggested that *Sporomusa* sp. could be acid-tolerant acetogens capable of activity in the pH range of 4.5–5.5. Additionally, under operating conditions of ORP = - 430 mV [11], acetogens could be the dominant species in the bacterial community structure [28].

### Archaeal 16S rDNA PCR-DGGE

**Fig 3:**
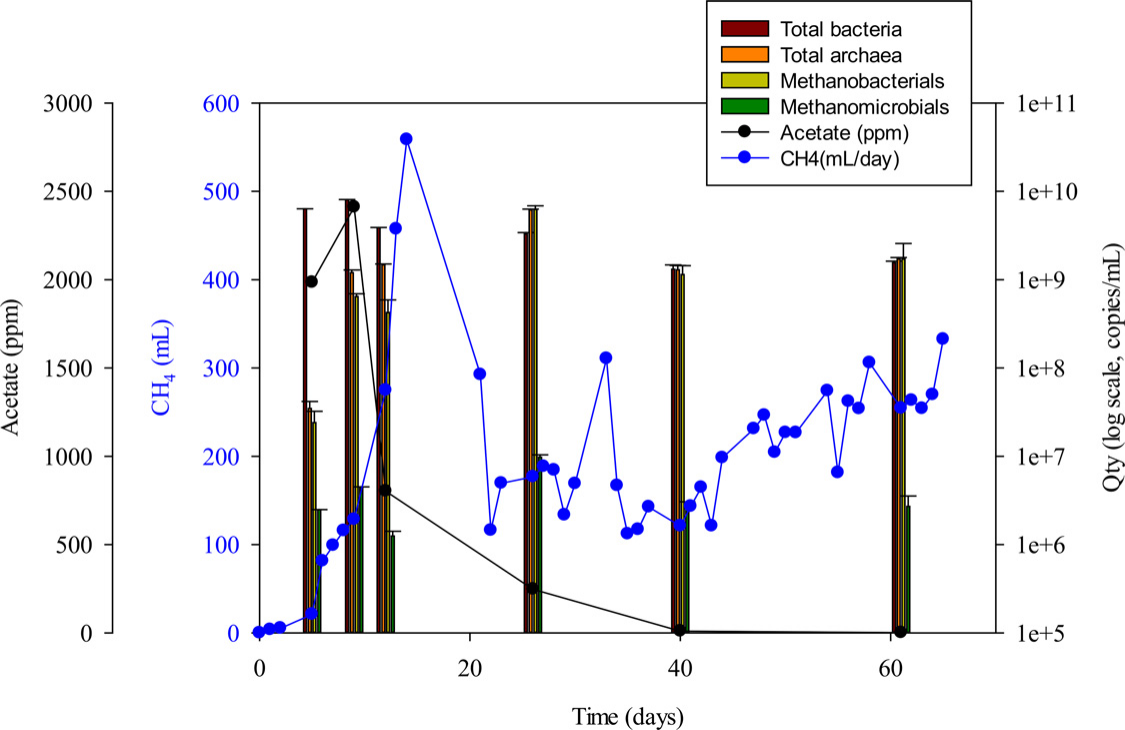
The profile of CH_4_ production (blue circle) and acetate concentration (black circle) co-plotted with the numbers of bacteria and archaea (bars). The results of real-time qPCR are shown as Qty (average quantity value) and standard deviations were shown as error bars.

The results of the archaeal 16S rDNA PCR-DGGE are shown in Fig 2C and 2D. Despite the analysis having been conducted only five days after inoculation with the anaerobic sludge, we found that the microbial community of the reactors was already established and dominant (Fig 2C). At acidic pH, *Methanosarcinaceae* and *Methanospirillum* were dominant among the methanogens (Fig 2D). *Methanospirillum* is a hydrogenotrophic methanogen [29], and *Methanosarcina* is an acetoclastic methanogen [30]. *Methanospirillum hungatei* used formate or H_2_ and CO_2_ as substrates for CH_4_ formation and growth [31]. *Methanosaeta concilii* was reported to use acetate and CO_2_ as carbon sources [32]. The volume of produced CH_4_ from 10 to 20 days was much more than that of the other periods (Fig 3), and the band M1-2, identified as *Methanosaeta concillii*, was detected with highest intensity in the lane of day 8 by DGGE. Therefore, both acetoclastic and hydrogenotrophic methanogen increased CH_4_ production in days 10–15. As shown in Fig 2C and 2D, hydrogenotrophic and acetoclastic methanogens initially coexisted, but only the hydrogenotrophic methanogens were still alive at the end of the experiment. The stable presence of hydrogenotrophic methanogens indicates that a bubbleless membrane-diffusion device was successfully transferred the gaseous phase substrate. This device overcame the poor solubility of H_2_ and afforded higher H_2_ utilization efficiencies with consequent energy savings. Additionally, the CH_4_ ratio of effluent gases reached 80–90% at day 7 and this ratio held until the end of operation [11, 33]. This result showed that CH_4_ production and microbial community structure became stable in the initial phase of operation.

### Real-time qPCR of bacteria and archaea

To quantify the archaea with a role in CH_4_ production, we used qPCR analysis with a specific primer to detect total archaea. Specifically, *Methanobacteriales*, representing hydrogenotrophic methanogens, and *Methanomicrobiales*, representing acetoclastic methanogens were tracked to discover their main mode of CH_4_ production. Bacterial numbers were also quantified with general bacterial primers (Table 3). The concentration of the total archaea ranged from 10^9^ to 10^10^ (copies/mL) during the operating periods of the reactors. All of the archaea, *Methanobacteriales*, and *Methanomicrobiales* had a similar pattern over time (Fig 3). Most of the archaea consisted of *Methanobacteriales*, but *Methanomicrobiales* were present at only levels of 0.01–1% (Fig 3). *Methanobacteriales* abundance dramatically increased during day 5, and the increased level was maintained afterwards. Although *Methanomicrobiales* abundance slightly increased temporarily from day 12 to 26, they returned to their initial concentration level over time (Fig 3).

The CH_4_ production was highest at 15 h (Fig 3), due to synergistic effects from both the hydrogenotrophic and acetoclastic methanogens. Produced acetic acid was quickly consumed during this period, as shown in Fig 3. Acetic acid concentrations increased initially but quickly decreased, and no acetic acid was detected after day 36. It is believed that acetate was produced by acetogens, such as *Sporomusa* sp. (shown in DGGE band in Fig 2B) and it was consumed by acetoclastic methanogens like *Methanosarcina* (Fig 2D). Acetate consumption led to the dominance of hydrogenotrophic methanogens (Fig 2C). We suggest that the pH drop inhibited acetogen growth and inhibited acetate production. In a previous study, when another reactor was operated at neutral conditions (pH = 7), acetate concentration was stable at 4–6 g /L [11], whereas the only acetate consumption was in the acidic reactor. Additionally, the qPCR result showed a decrease of bacterial numbers with increasing fermentation time, indicating that low pH inhibited the growth of acetate-producing bacteria.

We also observed CH_4_ consumption from day 14–21. Recently, methanogens were reported to have the ability to oxidize a small amount of CH_4_ anaerobically when CH_4_ was produced [34]. However, another study showed that the anaerobic oxidation of CH_4_ was not directly mediated by methanogenic bacteria [35]. The reduction of sulfate or nitrate was related to CH_4_ oxidation [36]. It is possible that the CH_4_ oxidation level was high at ~ day 15 from sulfate-reducing bacteria (the presence of nitrate reducing bacteria at ORP = - 430 mV being very unlikely [11]) and decreased as the acidophilic *Methanobacterium* sp. became established in and then dominated [37] the microbial community.

### Pyrosequencing of the microbial community

As shown in DGGE bands figure (Fig 2A and 2C), the DGGE did not show great microbial diversity and displayed only 2–4 bands. Some of the drawbacks of DGGE are the limited sequence information due to the small analyzed fragments (up to 500 bp) and its poor minor detection of small populations [38]. Additionally, it has been suggested that the DGGE for archaeal analysis should target each lower taxonomic groups [39]. Therefore, we used pyrosequencing for broad-based microbiome identification based on the sequencing-by-synthesis principle [40, 41].

When the reactor had stabilized without pH adjustment, the microbial community was analyzed with pyrosequencing. Pyrosequencing was expected to show more detailed information and correlations between the archaeal and bacterial community. The results showed that 97.1% of archaeal sequence reads were assigned at the species level, indicating that the analysis results could provide sufficient resolution for archaeal community analysis. In contrast, while most of the bacteria sequence reads were assigned at the species level, a considerable proportion of sequence reads (16%) were assigned at the order level. Therefore, bacteria were analyzed at both the order and the species levels.

#### Pyrosequencing of the archaeal community

**Fig 4:**
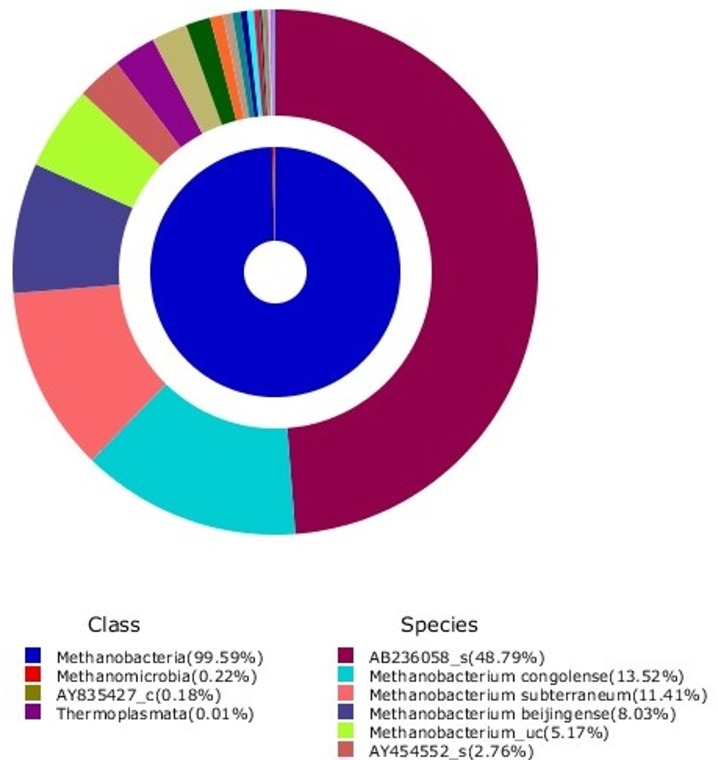
Abundance in the total community of major metanogenic species. Double pie chart shows major classes (inner) and species (outer). Mainly methanobacteria are present.

The taxonomic composition of methanogenic microbes is summarized in Table 4 (genus level) and Fig 4 (major classes (inner) and species (outer) in a double pie chart). The hydrogenotrophic methanogens *Methanobacteriales* dominated in the stabilized reactor. *Methanobacteriales* showed an abundance of 99% or more, and the acetoclastic methanogen *Methanosarcinales* were detected at only 0.22% of the total portion of the microbial community. The pyrosequencing method provided results assigned at the species levels. As shown in Fig 4, three strains dominated among the species. Among them, AB236058 (uncultured *Methanobacteriaceae*) had the largest abundance at 49%. *Methanobacterium congolense* and *M*. *subterraneum* both also had an abundance greater than 10%.

**Table 4:** Summary of major archaeal phylotypes of the microbial community.

Rank and Taxon				Genus % of total archaeal sequence reads	
Class	Order	Family	Genus	SUM (Ratio) %	Sum (Number)
*Methanobacteria*	*Methanobacteriales*	*Methanobacteriaceae*	*Methanobacterium*	99.04	7225
			*Methanobacteriaceae_uc*	0.41	30
			*Methanobrevibacter*	0.08	6
			*Methanosphaera*	0.03	2
			*Methanothermobacter*	0.01	1
		*Methanobacteriales_uc*	*Methanobacteriales_uc_g*	0.01	1
*Methanomicrobia*	*Methanosarcinales*	*Methanosaetaceae*	*Methanosaeta*	0.19	14
		*Methanosarcinaceae*	*Methanosarcina*	0.03	2
*Thermoplasmata*	Rice_cluster3_o	*Methanomassiliicoccus_f*	AF424770_g	0.01	1
AY835427_c	AY693811_o	AY693811_f	AY693811_g	0.14	10
			AY693811_f_uc	0.04	3

The strain AB236058 was detected in the CH_4_ producing process and observed in low H_2_ conditions [42]. *Methanobacteriaceae* became prevalent at low pH values [43]. *Methanonacterium congolense* is also a hydrogenotrophic methanogen. This strain uses CO_2_/H_2_ for cell growth but not acetate [44]. *Methanobacterium subterraneum* is representative of CH_4_ producing archaea and is capable of autotrophically growing in mineral medium without the addition of any organics [45]. *Methanobacterium beijingense* account for approximately 8% of the microbial community and has been reported to utilize H_2_/CO_2_ and formate [46]. A microbial community producing CH_4_ by hydrogenotrophy was developed without additional pH control. As previously stated, DGGE and real time PCR showed similar results with the pyrosequencing.

#### Pyrosequencing of the bacterial community

**Fig 5:**
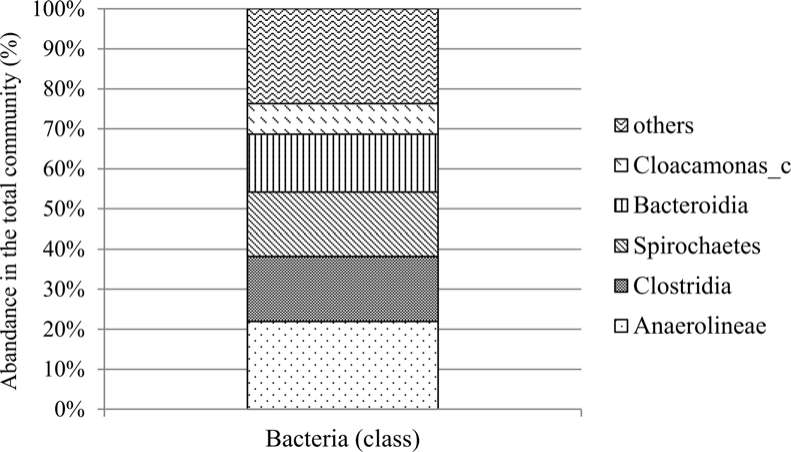
The distribution of major classes of the bacterial community. Diverse bacteria were present, and it was expected that they played a role in acetate consumption.

The taxonomic composition of the bacterial community is summarized in Table 5 (genus level) and Fig 5 (species level). Kim *et al*. reported that the methanogenic community shifted from acetoclastic methanogens to a hydrogenotrophic community, which is accompanied by an increase in the population of *Firmicutes*, in particular of *Clostridia*, in the bacterial community [47]. Thus, the stable bacterial community for CH_4_ production was evaluated in terms of how it shifted. As shown in the Table 5, most bacterial sequence reads were assigned as uncultured strains at the genus level. Among them, AH009469 had an abundance of 20.58%. This strain belongs to *Anaerolinaceae*. Some strains of *Anaerolinaceae* are known to grow with hydrogenotrophic methanogens [33]. In addition, strains of *Anaerolinaceae* have been detected as a digester of CH_4_ gas [48]. Next, AY214182 belonging to *Spirochaetaceae* had an abundance of 15.68% in the microbial community. Chartrain and Zeikus reported that *Spirochaetaceae* have been found to degrade ethanol to acetate in the presence of H_2_-consuming methanogens [49, 50]. The strain assigned to EF198044 had an abundance of 8%. This strain was uncultured and detected in the mesophilic and thermophilic phenol-degrading methanogenic consortia [51]. At the class level, *Clostridia* showed an abundance of 17%. Clostridia are known as strong H_2_ producers [52]. In addition, *Clostridia* can produce organic acids such as acetic acid by degrading organic substances. These characteristics of the microbial community would affect the methanogenic consortia.

**Table 5:** Summary of major bacterial phylotypes of the microbial community.

Rank and taxon					Genus % of total bacterial sequence reads	
Phylum	Class	Order	Family	Genus	% Sum (Ratio)	Sum (Number)
*Chloroflexi*	*Anaerolineae*	*Anaerolinaeles*	*Anaerolinaceae*	AJ009469_g	20.58999	2066
*Spirochaetes*	*Spirochaetes_c*	*Spirochaetales*	*Spirochaetaceae*	AY214182_g	15.68667	1574
OPB7	OPB7_c	OPB7_o	OPB7_f	EF198044_g	8.18218	821
*Cloacamonas_p*	*Cloacamonas_c*	*Cloacamonas_o*	*Cloacamonas_f*	*Cloacamonas*	7.37493	740
*Firmicutes*	*Clostridia*	DQ887962_o	JF417924_f	EU878324_g	7.25533	728
		*Clostridiales*	*Tissierella_f*	*Tissierella*	2.65099	266
			*Thermohalobacter_f*	*Proteiniborus*	1.67431	168
		*Thermaerobacter_o*	JF417922_f	JF417922_g	1.25573	126
*Firmicutes*	AB476673_c	AB476673_o	AB476673_o_uc	AB476673_o_uc_g	5.23221	525
*Bacteroidetes*	*Bacteroidia*	*Bacteroidales*	*Bacteroidales_uc*	*Bacteroidales_uc_g*	8.16225	819
			*Porphyromonadaceae*	*Petrimonas*	1.2059	121
		*Bacteroidia_uc*	*Bacteroidia_uc_f*	*Bacteroidia_uc_g*	1.03648	104
FN436175_p	FN436175_c	FN436175_o	FN436175_f	FN436175_g	2.33207	234

**Fig 6:**
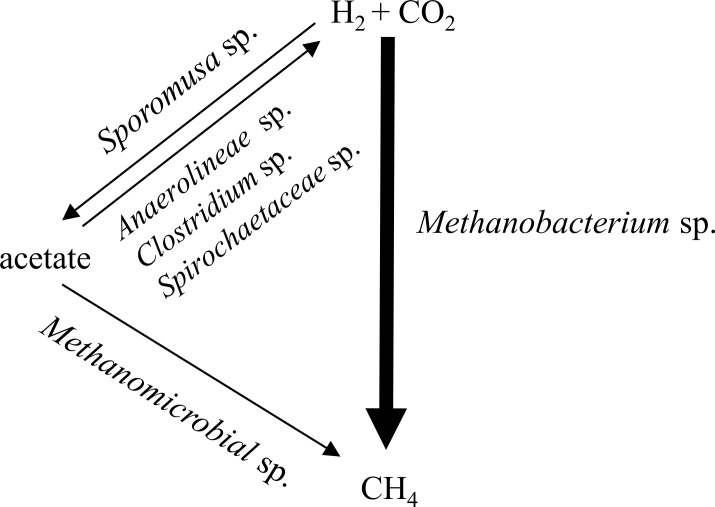
The microbial community development in acidic methanogenic reactor. The acetate was produced by acetogens from H_2_ and CO_2_. Acetate was used by bacteria and H_2_ and CO_2_ were regenerated. Finally, acetate was limited, but H_2_ continued to be available, and hydrogenotrophic methanogens were harvested.

Both DGGE and pyrosequencing analysis showed the enrichment of hydrogenotrophic methanogen in Hf-MBfR without pH control. The time profile of DGGE bands did not show substantial diversity in the microbial community, but pyrosequencing indicated the enrichment of hydrogenotrophic methanogens in the whole microbial community even though it supplied less information about bacterial community at the species level. Additionally, qPCR data evinced both the enrichment of hydrogenotrophic methanogens and the pH inhibition on bacterial growth. These analyses were helpful in understanding the relationship between the bacteria and the methanogens in the Hf-MBfR (Fig 6). The acidic methanogenic reactor showed hydrogenotrophic methanogen enrichment, and low pH seems to have inhibited acetogen growth. Limited acetate production led to the growth of hydrogenotrophic methanogens rather than acetoclastic methanogens. Low pH inhibited bacterial growth, especially of acetogens as acetate suppliers to acetoclastic methanogens, and enriched hydrogenotrophic methanogens (Fig 6).

## Conclusions

In this study, we investigated the characteristics of the microbial community in a hollow-fiber membrane biofilm reactor (Hf-MBfR), focusing on the presence of hydrogenotrophic methanogens that convert supplied CO_2_ and H_2_ into CH_4._ The reactor was operated under acidic conditions, and the microbial community was analyzed with 16S rDNA PCR-DGGE, real-time qPCR, and pyrosequencing. The results showed a stable microbial community was established relatively early in the fermentation, and this community was enriched with hydrogenotrophic methanogens (99.6% of archaea). Therefore, the high conversion efficiency of CO_2_ to CH_4_ was induced by the enrichment of hydrogenotrophic methanogens by the acidic operation of the Hf-MBfR, which mitigated the disadvantage of H_2_ as electron donors (less soluble in water, explosive gas easily released from air diffuser). The community structure showed that the Hf-MBfR properly supplied the hydrogen for hydrogenotrophic methanogen. Additionally, the result suggested that the acidic operation of Hf-MBfR inhibited acetogens and led to the enrichment of hydrogenotrophic methanogens, achieving a high conversion ratio of CO_2_ to CH_4_.

## Supporting Information

S1 Fig The bacterial community structure of initial sludge and enrichment culture in Hf-MBfR at the phylum level. Initial sludge indicates the microbial community in the inoculum, and Hf-MBfR indicates the microbial distribution by phylum after the enrichment of hydrogenotrophic methanogen. The final microbial community in the Hf-MBfR appears very different from the inoculum. ETC means minor components (cut off was 1.0% of total abundance).
(DOCX)

S1 File Krona_charts_supplemental.zip. Interactive Krona HTML5 hierarchical and double pie chart of bacterial, archaeal community profiles.
(ZIP)
