# Supplementary material for: Analysis of the Microbial Community in an Acidic Hollow-Fiber Membrane Biofilm Reactor (Hf-MBfR) Used for the Biological Conversion of Carbon Dioxide to Methane
Source: PLoS One. 2015 Dec 22;10(12):e0144999. doi: 10.1371/journal.pone.0144999 (PMC4687861; doi:10.1371/journal.pone.0144999)
Supplement: S1 Fig — Initial sludge indicates the microbial community in the inoculum, and Hf-MBfR indicates the microbial distribution by phylum after the enrichment of hydrogenotrophic methanogen. The final microbial community in the Hf-MBfR appears very different from the inoculum. ETC means minor components (cut off was 1.0% of total abundance). (DOCX) [file pone.0144999.s001.docx]

**S1 Fig. The bacterial community structure of initial sludge and enrichment culture in Hf-MBfR as shown with phylum.**

Initial sludge indicates the microbial community in the inoculum and Hf-MBfR showed microbial distribution as the phylum after enrichment of hydrogenotrophic methanogen. It suggests that final microbial community in Hf-MBfR was much different with initial inoculum. ETC means minor components (cut off was 1.0 %).
