# Supplementary material for: Analysis of the Microbial Community in an Acidic Hollow-Fiber Membrane Biofilm Reactor (Hf-MBfR) Used for the Biological Conversion of Carbon Dioxide to Methane
Source: PLoS One. 2015 Dec 22;10(12):e0144999. doi: 10.1371/journal.pone.0144999 (PMC4687861; doi:10.1371/journal.pone.0144999)
Supplement: S1 File — Interactive Krona HTML5 hierarchical and double pie chart of bacterial, archaeal community profiles. (ZIP) [file pone.0144999.s002.zip › charts_supplemental/Krona chart_archaea.html]

Javascript must be enabled to view this page.

members
magnitude

C\_018040.krona

7295

7295

7295

16

16

14

14

1

9

4

2

2

2

7265

7265

1

7264

2

1

1

6

2

4

7225

586

986

3559

42

37

111

2

201

832

58

8

1

11

26

29

377

1

4

160

2

191

1

30

1

1

13

13

13

10

10

3

1

1

1

1

1
