# Supplementary material for: Analysis of the Microbial Community in an Acidic Hollow-Fiber Membrane Biofilm Reactor (Hf-MBfR) Used for the Biological Conversion of Carbon Dioxide to Methane
Source: PLoS One. 2015 Dec 22;10(12):e0144999. doi: 10.1371/journal.pone.0144999 (PMC4687861; doi:10.1371/journal.pone.0144999)
Supplement: S1 File — Interactive Krona HTML5 hierarchical and double pie chart of bacterial, archaeal community profiles. (ZIP) [file pone.0144999.s002.zip › charts_supplemental/Krona chart_bacteria.html]

Javascript must be enabled to view this page.

members
magnitude

C\_018240.krona

10034

10034

834

834

4

830

7

823

821

817

4

2

18

18

1

17

4

6

5

1

1

7

6

1

1

1

1

1

1

1

1460

3

3

3

2

1

1

5

1452

1348

7

5

4

1

1

1

1

57

13

5

8

42

40

2

2

36

36

36

6

5

5

1

1

280

10

121

30

19

4

68

14

9

5

2

2

133

2

1

16

114

3

3

3

2

2

1

2

2

819

135

12

14

13

1

84

84

25

15

10

104

1

1

1

1

1

1

55

55

55

2

2

49

6

43

1

42

4

9

9

9

9

7

4

1

2

2

1

1

1

1

1

52

1

51

50

50

22

17

5

20

2

18

8

1

2

2

2

2

2

20

10

10

10

9

9

1

10

6

6

4

172

9

4

4

4

5

91

31

31

60

72

2

61

45

36

10

26

17

9

1

8

9

9

3

1

1

5

5

6

6

2

1

1

1

2

2

2

1

1

1

1

1

1

3

3

2

2

2

1

1

1

1

1

1

4

4

1

1

3

3

3

79

4

2

1

1

1

1

1

2

1

1

1

1

4

1

1

1

2

2

1

1

1

1

1

1

1

1

24

1

1

1

1

21

2

2

2

1

1

1

2

2

2

1

1

1

15

5

5

1

1

1

1

7

4

3

1

1

2

2

1

1

1

46

1

1

1

1

43

3

40

1

1

16

22

22

1

1

2

263

263

4

259

9

250

234

225

9

16

769

769

769

1

768

740

7

66

655

7

5

28

8

7

5

2

2

1

1

1

1

1

1

1

6

2

2

2

4

4

4

1

1

2

1

1

1

1

1

1

1607

1

1606

16

1590

1587

1574

254

1320

13

1

1

1

2

2224

1627

30

10

3

1

2

7

20

1

1

837

804

728

36

666

26

14

62

7

15

40

8

2

2

6

25

3

2

1

1

1

1

132

4

128

2

126

2

47

3

74

22

1

1

601

1

1

275

2

2

7

266

230

35

1

5

5

3

1

1

1

1

7

7

1

1

1

3

1

1

2

170

168

166

2

2

8

1

7

7

2

2

17

1

1

1

1

8

3

3

1

1

2

2

1

1

24

2

1

1

1

1

21

3

16

2

1

1

6

5

1

2

2

1

21

3

3

4

2

1

1

12

12

4

4

49

3

3

3

3

3

3

3

1

1

1

2

2

1

1

3

2

2

2

36

36

36

36

24

12

553

527

2

2

525

26

2234

31

13

2

2

2

11

1

1

6

4

1

1

3

1

2

1

1

4

4

4

4

1

1

1

2202

2199

1

1

1

9

2189

3

3

14

58

7

51

2

2

10

1

9

14

14

1

1

21

20

1

2066

18

12

1

12

24

2

1991

6

1

1

1

1

2

83

83

21

21

13

4

1

8

8

62

39

5

1

1

1

1

32

27

5

11

7

2

1

4

1

3

3

12

12

12

6

1

5

3

3

3

3

2

2

2

2

2

2

2

2

2

56

56

1

55

6

13

9

9

2

2

2

2

36

36

2

2

2

1

1

1

3

3

3

2

2

1
